# Evaluation of Constructing Care Collaboration - nurturing empathy and peer-to-peer learning in medical students who participate in voluntary structured service learning programmes for migrant workers

**DOI:** 10.1186/s12909-019-1740-6

**Published:** 2019-08-08

**Authors:** DYE Sin, TCT Chew, T. K. Chia, J. S. Ser, A. Sayampanathan, GCH Koh

**Affiliations:** 1 Yong Loo Lin School of Medicine, National University of Singapore, Singapore, Singapore; 2 Saw Swee Hock School of Public Health, National University of Singapore, Singapore, Singapore

**Keywords:** Medical education, Service learning, Peer-to-peer learning

## Abstract

**Background:**

Experiential learning through service provides opportunities to nurture and practice empathy. Of growing concern, studies showed significantly decreased empathy scores as students progress through medical school. Additionally, peer-to-peer learning provides an effective way for students to learn. Constructing Care Collaboration (CCC) is a student initiated, structured-service-learning-program that promotes the development of empathy and peer-to-peer teaching. CCC is conducted in cycles of 6 sessions. This is a mixed methods study that explores the effectiveness of CCC as a service learning platform in developing student participants’ empathy, social and cultural competencies, communication skills and peer-to-peer teaching skills, ultimately aiming to promote a culture of serving the underprivileged.

**Methods:**

The study comprised of a self-administered quantitative questionnaire and qualitative interviews. Both evaluated if CCC participation developed volunteers’ social-awareness, cultural competency, communication, confidence and motivation to teach their peers.

**Results:**

Quantitative data were collated from 38 completed student volunteers’ questionnaires. Volunteers generally agreed CCC improved social-awareness and cultural competency. It increased confidence of participants in approaching migrant-workers, communicating with people from different social backgrounds, and promoted a culture of peer-to-peer teaching.

Thematic analysis of 17 interviews was conducted. Themes identified include: increased empathy towards migrant-workers, improved communication skills, and identifying benefits and challenges in peer-to-peer teaching.

**Conclusion:**

From the quantitative and qualitative information gathered, CCC has been shown to be effective in nurturing participants’ self-reported empathy, cultural competence, communication skills and improved attitude towards peer-to-peer teaching. Given its effectiveness, CCC can be adopted as a model for structured service-learning.

**Electronic supplementary material:**

The online version of this article (10.1186/s12909-019-1740-6) contains supplementary material, which is available to authorized users.

## Background

Community work has been shown to harness great benefits for the volunteer. Not only does it confer numerous health benefits and improve psychological well-being [1], it inculcates a greater sense of appreciation and ownership of one’s community, and an ethics of service [2].

There are numerous structured volunteering programmes worldwide, which contribute greatly to medical education when tailored to allow volunteers to meet certain learning objectives. While there are many forms of service learning, they generally share common traits of community service or experiential learning, and introspective reflection [3]. In the healthcare setting, volunteering not only allows medical students to learn [4] and cultivate ideals like altruism [5], it also promotes a culture of community outreach [6, 7]. Participants of service learning programmes report improved attitudes towards patients [8]. Several reports on service learning programs attribute these benefits to the exposure to a culturally different community that they otherwise would not have interacted with [9], and to structured reflection of the experience [10]. Cultural competence is often defined in literature as having the attitudes, behaviours and practices to provide effective healthcare, taking into account the background, beliefs and needs of patients from different cultures [11, 12]. Cultural awareness is the knowledge and appreciation of these backgrounds, beliefs and needs and this is essential for culturally competent healthcare [11–13]. The experience in CCC may hence prepare medical students for work in culturally diverse settings [14].

In Singapore, there are numerous structured volunteering programmes. The pedagogical value of some of these volunteering programmes that cater to the local Singaporean population have been evaluated [15]. However, volunteer programmes that administer to the needs of the foreign population in Singapore, such as the Constructing Care Collaboration (CCC), have not been evaluated for its value in medical education. This paper thus seeks to evaluate the pedagogical value of such a volunteer programme.

### Programme description

CCC is a pioneering student-led service learning initiative that was started in 2013 to involve medical students in learning about and serving migrant communities. As of June 2017, Singapore employed 296,700 construction workers under its work permit pass [16]. The majority of migrant workers in construction come from India, Bangladesh and China, and draw a monthly salary of S$700–1200 (US$510–876). As foreigners in Singapore do not receive subsidies for healthcare at public institutions, some workers find it difficult to access care due to cost constraints [17]. CCC complements charity community clinics that provide subsidized healthcare for migrant labour workers. These clinics are operated by Non-Governmental Organizations (NGOs). Volunteer doctors, allied health care professionals, medical students and lay people staff these clinics.

CCC engages medical students to volunteer at charity community clinics in either the dormitories of the worker or a popular social gathering place of the workers. This is with the aim of both assisting and serving in two community clinics (Caring Clinic (non-dormitory based) and Penjuru Clinic (dormitory based)). With such exposure, CCC hopes to develop empathy amongst medical students which requires time and cannot always be effectively nurtured in the basic medical curriculum. Through this platform, CCC also aims to promote a culture of peer-to-peer learning which is not commonly observed in formal medical education activities.

Medical students (volunteers) help to take the medical history of the migrant workers and seek to understand the lives of migrant workers beyond their medical condition. Volunteers, who have the benefit of spending more time with the patient, would then present the patient’s medical history to volunteer doctors and help to clarify any issues raised during the consultation. Doctors will confirm pertinent points of the history, perform a physical examination, diagnose, prescribe treatment and advise migrant workers. Migrant workers then collect medications and pay a nominal fee of S$5 regardless of medication prescribed. Due to limited resources, only basic procedures and medication are offered by the charity clinics. Patients requiring more complex procedures, expensive medication, biochemical investigations or imaging are referred to the hospital. Medical students may occasionally accompany the migrant workers to the hospital.

Medical student volunteers are placed into groups and attend a clinic session once a month for a total of 6 months. Each session is structured to focus on a certain aspect of the lives of a migrant worker, with each session encouraging volunteers to gain progressively deeper insights into migrant workers’ lives. Six sessions make up one cycle of CCC.

At the start of each session, group leaders (senior medical students from clinical years) brief their group on the day’s focus and task (e.g. finding out the migrant workers’ motivations to come to Singapore). Medical student volunteers are then paired, 1 senior (clinical years) with 1–2 juniors (pre-clinical), to allow seniors to guide and teach juniors. Volunteers will then follow the workflow of the clinic. At the end of every session, group leaders lead a sit-down discussion on the sessions’ focus, sharing volunteers’ experiences as well as teaching relevant medical knowledge. Migrant workers differ greatly from most medical students in terms of background, circumstance and socioeconomic status. Through observations, reflections and engagement during CCC, medical students learn to better relate to and understand migrant workers and all individuals in their care. Formal structured discussions and informal sharing sessions amongst peers also aim to cultivate the practice of peer-to-peer teaching amongst medical students.

### Logistics

CCC is a student initiative by an Asian undergraduate medical school, the Yong Loo Lin School of Medicine, National University of Singapore (NUS Medicine). All medical students from NUS Medicine including pre-clinical (years 1–2) and clinical (years 3–5) are invited to volunteer for CCC. Medical Student Volunteers are selected to achieve a clinical to pre-clinical student ratio of roughly 1:1–2 and are paired in such a ratio. In the case of an overwhelming response, volunteers are selected at random. In NUS, each medical year consists of 300 students. Out of this, an estimate of about 60–80 volunteer for CCC (as of 2017). Volunteers do not receive any academic credits for their service.

All volunteers are briefed on the workflow of the clinic and the structure of the program before each cycle begins. Equipment such as stethoscopes, torches, sphygmomanometers and clerking sheets are often brought by volunteer students or doctors. Transport to and from the clinics are self-arranged and paid for by volunteers.

### Aim

This study aims to both quantitatively and qualitatively explore, through participants’ self evaluation and reflection, if and how CCC was beneficial as a service learning platform, in developing student participants’ empathy, social and cultural competence, communication skills and peer-to-peer teaching skills. This study also aims to evaluate how CCC may inculcate students with a holistic and global perspective of serving other underprivileged sectors of society, beyond the local population in Singapore.

## Methods

This is a mixed method study that comprises of a self-administered quantitative questionnaire and qualitative interview. Participants for the study were recruited from medical student volunteers who had finished at least 1 cycle of volunteering with CCC. Mixed methods was chosen to maximize the unique strengths and limitations of both qualitative and quantitative methods which are discussed below.

### Quantitative questionnaires

In an attempt to assess the quality of CCC as a service learning platform, we adopted questions from the Fund for the Improvement of Postsecondary Education (FIPSE) survey instruments [18]. We adopted the Ability Scale which was designed for an Asian population in Taiwan [19] as it subjectively evaluates skills acquired from service learning projects and also allows for comparison with other projects previously studied. The questionnaire is a self-evaluation of participants’ gains in the areas of 1) Leadership skills, 2) Communication Skills, 3) Teamwork, 4) Ability to see consequences, 5) Critical thinking skills, 6) Ability to identify social issues, 7) Action skills, 8) Gaining of knowledge, 9) Application of knowledge. Additional questions were designed to obtain participants’ opinions on peer-to-peer teaching by their fellow student seniors in CCC and to assess specific gains in skills to communicate with migrant workers (Additional file 1). We attempted to apply statistical analysis, but a lower than expected response rate made it difficult to do so. Nonetheless, it provided information which the authors felt meaningful to evaluate the program and share.

### Qualitative interviews

The qualitative interviews allowed an in-depth exploration of participants’ views of CCC. Participants were engaged in the form of semi-structured open-ended questions regarding CCC’s impact on their empathy, social and cultural competence and communication skills as well as questions on the curriculum of each cycle and the culture of peer-to-peer teaching (Additional file 2). Participants whom were interviewed also completed the quantitative questionnaires. Participants to be interviewed were selected to ensure adequate representation of volunteers from pre-clinical and clinical years. Participants were interviewed by the authors of this article (SYE, TCLT, TCK, SJS). Each interview was conducted face to face in person in a private environment, with each interview lasting approximately 30 min. The interviews were transcribed for later analysis and coding, by the authors.

### Recruitment of participants

All participants recruited were students from NUS Medicine. There are total of 5 cohorts (1500 students) each year, ranging from first year students to final year students in their 5th year. CCC recruits an estimated 60–80 students in each cycle. A total of 38 participants were recruited to this study (*n* = 38 for the quantitative survey of which 17 participated in the qualitative survey).

### Inclusion criteria

Members of CCC who were present at the final session of their cycle in 2015–16 were invited to participate in the quantitative survey. This consisted of participants who were volunteering on the programme for the first time, and those who had also volunteered in previous cycles. Volunteers of CCC who had completed at least 1 cycle were invited to participate in a qualitative interview on their experience volunteering with CCC. Volunteers recruited included participants who were regular volunteers and participants who were group leaders or executive committee members.

### Ethics approval

This study was approved by the Institutional Review Boards of National University of Singapore (IRB Ref: B-15-142). Participants who answered the anonymous self-administered quantitative questionnaire provided verbal consent. Participants who answered qualitative interviews provided written consent. We distributed a participant information sheet to all participants.

### Data analysis

We performed all quantitative analyses using IBM SPSS Statistics software Version 23.0 (IBM Co., Armonk, New York, US), Chi squared test and Fisher’s exact test. Statistical significance was set at *P* < 0.05. Quantitative data from the 4 point likert scale questionnaire was binarised by taking “agree” and “unsure but tend to agree” as 1 and the other 2 options as 0. Option 5 “Do not understand the statement” was removed from analysis. We recorded and transcribed ad verbatim all qualitative interviews. After which, they were coded using thematic analysis as outlined by Braun and Clarke [20], independently by 4 of the authors (SYE, TCLT, TCK, SJS). Transcripts were first briefly reviewed to discern ideas brought up by participants. Recurrent/similar ideas were consolidated. The concepts/ideas were then grouped into subthemes, which were subsequently grouped under the various themes: empathy, social awareness, cultural competence, peer-to-peer teaching and the CCC topics. The frequency which concepts and subthemes occurred were counted and analysed to determine which subthemes were more prominent. Qualitative data collection ceased once data saturation was reached at the 15th interview, but 2 more interviews were conducted to confirm that there were no further new themes that could be elicited.

## Results

We approached all volunteers at the last session with a 100% response rate. There was an attendance rate of 52.8% at the last session. Table 1 details the demographics of participants of the quantitative survey. There was an approximately equal distribution of participants across gender and age group. With regards to demographics of participants of the qualitative interviews, there was a similar distribution of participants across gender, age group, year of study, clinic attended and role in CCC.

**Table 1:** Outline of sessions

Session	Details
1 – Commitment	Volunteers reflect on their preconceived impressions, develop relationships and meaningful conversations with migrant workers, and compare the demographics, background and experiences with their impressions.
2 – Compassion	Volunteers explore migrant workers’ journeys and reasons for traveling to Singapore and reflect on the problems faced by the migrant worker they clerked.
3 – Communication	Volunteers focus on difficulties faced when taking history from the migrant workers, reflect on solutions and consider problems faced by migrant workers in a typical public healthcare setting.
4 – Care	Volunteers explore migrant workers’ ideas, concerns and expectations and reflect on how they differ from their local counterpart.
5 – Concern	Volunteers reflect on the general health and wellbeing of migrant workers, and the resources, or lack thereof, available.
6 – Continuity	Volunteers reflect on their initial motivations for volunteering, what they have learnt, and how they would proceed thereon.

### Quantitative study

In general, the majority of participants reported that volunteering in CCC benefited them across all 9 domains of the FIPSE instrument as well as the 2 additional domains of communication skills specific to interacting with migrant workers and peer-to-peer-teaching skills. (Table 2).

**Table 2:** Demographics of participants

Baseline characteristics of study participants	Quantitative Survey Demographics, *n* (%) (*N* = 38)	Qualitative Interview Demographics *n* (%) (*N* = 17)
Gender		
Males	20 (52.6)	7 (41.2)
Females	18 (47.4)	10 (58.8)
Medical Year		
Preclinical	26 (68.4)	10 (58.8)
Clinical	12 (31.6)	7 (41.2)
Age		
< 21 Years old	18 (47.4)	5 (29.4)
21 years old and above	20 (52.6)	12 (70.6)
Ethnicity		
Chinese	37 (97.4)	14 (82.4)
Non-Chinese	1 (2.6)	3 (17.6)
Number of Cycles		
1 Cycle	27 (77.1)	9 (52.9)
2 or more cycles	11 (28.9)	8 (47.1)
Number of sessions attended		
6 or less	29 (76.3)	9 (52.9)
More than 6	9 (23.7)	8 (47.1)
Clinic attended		
Penjuru Clinic	22 (57.9)	9 (50.0)
Caring Clinic	15 (37.5)	9 (50.0)
Roles in CCC		
Participant	28 (73.7)	10 (58.8)
Leader	10 (26.3)	7 (41.2)

There were no significant associations between gender, medical year, number of cycles, clinic attended or role in CCC with all areas except for the following, taking the value of (*p* < 0.01) (Table 3). A greater percentage of participants who volunteered in non-dormitory based community clinics reported benefit in thinking about the future consequence for patients compared to participants from clinic situated in dormitories (100% vs 60.9%), OR = 0.61 (95% CI 0.44, 0.85) (*p* = 0.006) Participants in leadership positions were also more likely to report benefit in enhancing their understanding of the use of public health measures in resource-poor settings as compared to participants who were regular volunteers. (100% vs 66.7%), OR = 0.6 (95% CI 0.49, 0.85) (*p* = 0.038).

**Table 3:** Responses for quantitative survey

Volunteering in CCC has helped me…	Total No who agree with the statemen. (% of responses)	Gender Male: female Unadjusted odds ratio (OR) (95% confidence interval)			Preclinical: Clinical Unadjusted odds ratio (OR) (95% confidence interval)			Single cycle: multiple cycles Unadjusted odds ratio (OR) (95% confidence interval)			Penjuru: Caring clinic Unadjusted odds ratio (OR) (95% confidence interval)			Participant: Leader Unadjusted odds ratio (OR) (95% confidence interval)		
		OR	95% CI	*P*	OR	95% CI	*P*	OR	95% CI	*P*	OR	95% CI	*P*	OR	95% CI	*P*
Leadership Skills																
Feel more responsible for others in the Community	35 (92.1)	1.20	0.98–1.48	0.10	0.89	0.77–1.02	0.54	5.78	0.47–71.6	0.20	0.87	0.74–1.02	0.26	1.44	0.12–17.9	1.00
Improve my leadership skills	26 (68.4)	0.43	0.10–1.79	0.31	0.63	0.14–2.93	0.71	0.38	0.07–2.11	0.44	0.39	0.09–1.77	0.29	0.17	0.02–1.55	0.12
Communication Skills																
Participate in community affairs	30 (78.9)	1.14	0.24–5.44	1.00	0.24	0.03–2.28	0.39	0.78	0.13–4.62	1.00	0.44	0.08–2.52	0.44	0.33	0.04–3.12	0.65
Further develop communication, listening and negotiation skills	36 (94.7)	1.12	0.07–19.30	1.00	2.27	0.13–39.70	0.54	0.93	0.83–1.03	1.00	1.57	0.09–27.2	1.00	0.93	0.84–1.03	1.00
Teamwork																
Think more of others	34 (91.9)	2.25	0.19–27.20	0.61	0.88	0.76–1.02	0.54	1.39	0.11–17.24	1.00	0.86	0.73–1.020	0.26	0.89	0.78–1.02	0.55
Appreciate teamwork and co-operation among peers	37 (97.4)	1.06	0.95–1.18	0.47	0.96	0.89–1.04	1.00	1.10	0.91–1.33	0.29	0.96	0.88–1.04	1.00	0.96	0.90–1.04	1.00
Be more tolerant of different people	36 (94.7)	1.12	0.07–19.30	1.00	2.27	0.13–39.70	0.54	0.93	0.83–1.03	1.00	1.57	0.09–27.2	1.00	0.93	0.90–1.03	1.00
Respect different opinions	32 (84.2)	0.50	0.08–3.13	0.66	1.10	0.17–7.03	1.00	0.44	0.05–4.27	0.65	0.26	0.03–2.46	0.37	1.50	0.23–9.80	0.64
Compromise	31 (83.8)	1.21	0.21–6.99	1.00	0.36	0.04–3.52	0.64	0.42	0.04–4.09	0.65	0.79	0.13–5.01	1.00	0.78	0.64–0.95	0.16
Better Comprehend the moral and ethical issues in health care	32 (84.2)	1.13	0.20–6.49	1.00	0.38	0.04–3.69	0.64	0.44	0.05–4.27	0.65	0.73	0.12–4.59	1.00	0.51	0.05–5.00	1.00
Ability to see consequences																
Think more about the future	29 (80.6)	0.70	0.13–3.70	1.00	0.76	0.12–4.64	0.57	0.37	0.04–3.54	0.65	0.67	0.49–0.90	0.03	0.37	0.04–3.54	0.65
Critical thinking skills																
Think more critically	36 (94.7)	1.13	0.96–1.33	0.22	0.92	0.83–1.03	1.00	2.60	0.15–45.7	0.50	0.91	0.81–1.04	0.51	0.93	0.84–1.03	1.00
Ability to identify social issues																
Better Identify social issues and concerns	34 (89.5)	1.13	0.14–8.94	1.00	0.68	0.07–7.49	1.00	0.85	0.73–1.00	0.30	1.62	0.20–12.9	1.00	0.86	0.74–1.00	0.56
Action Skills																
Take more action	32 (86.5)	1.70	0.25–11.59	0.66	0.48	0.05–4.806	1.00	0.639	0.06–6.52	1.00	0.32	0.03–3.21	0.63	0.82	0.68–0.98	0.30
Build confidence & take on new responsibilities	33 (86.8)	1.80	0.27–12.20	0.65	0.50	0.05–5.03	1.00	1.78	0.25–12.4	0.62	1.03	0.15–7.00	1.00	0.82	0.69–0.98	0.30
Gaining of knowledge																
Appreciate and identify better gaps or deficiencies in the healthcare system	31 (81.6)	0.80	0.15–4.18	1.00	0.84	0.14–5.10	1.00	0.74	0.59–0.93	0.08	1.19	0.23–6.26	1.00	0.75	0.61–0.93	0.16
Better Appreciate my own health, living condition	35 (94.6)	1.19	0.07–20.5	1.00	0.92	0.82–1.03	1.00	0.93	0.83–1.03	1.00	0.91	0.80–1.04	0.51	0.93	0.83–1.03	1.00
Improve my medical knowledge	35 (92.1)	2.38	0.20–28.70	0.60	1.09	0.09–13.3	1.00	1.25	0.10–15.4	1.00	0.87	0.74–1.02	0.26	0.89	0.79–1.02	0.55
Enhance my understanding of the use of public health measures in resource-poor settings	28 (73.7)	1.15	0.27–4.90	1.00	0.45	0.08–2.54	0.45	0.20	0.02–1.82	0.23	1.03	0.24–4.50	1.00	0.64	0.49–0.85	0.04
Application of knowledge																
Improve my clinical diagnostic skills	34 (89.5)	1.13	0.14–8.94	1.00	2.40	0.30–19.5	0.58	0.80	0.74–8.65	1.00	0.48	0.05–5.06	1.00	0.86	0.74–0.997	0.56
Apply what I learnt in medical school	35 (92.1)	2.38	0.20–28.7	0.60	1.09	0.09–13.3	1.00	1.25	0.10–15.4	1.00	0.87	0.74–1.02	0.26	0.89	0.79–1.02	0.55
Communication Skills - with migrant workers specifically																
Increase my confidence in approaching migrant workers	35 (92.1)	0.53	0.04–6.39	1.00	1.09	0.09–13.3	1.00	0.89	0.78–1.02	0.54	0.75	0.06–9.08	1.00	0.89	0.79–1.02	0.55
Increase my confidence in talking to people from different social backgrounds.	36 (94.7)	1.12	0.07–19.3	1.00	2.27	0.13–39.7	0.54	0.93	0.83–1.03	1.00	1.57	0.09–27.2	1.00	0.93	0.84–1.03	1.00
Peer-to-Peer teaching skills - Volunteering in CCC has helped me/I feel																
Increase my confidence in teaching my peers	32 (84.2)	0.50	0.08–3.13	0.66	1.10	0.17–7.03	1.00	0.44	0.05–4.27	0.65	0.73	0.12–4.59	1.00	0.79	0.65–0.95	0.17
Increase my knowledge from what was taught by my peers during CCC	34 (89.5)	0.33	0.03–3.53	0.61	8.33	0.77–90.8	0.16	0.80	0.07–8.65	1.00	0.48	0.05–5.06	1.00	0.86	0.74–1.00	0.56
My experience in peer to peer teaching at CCC has enabled me to learn more from similar modes of peer-to-peer teaching (outside of CCC)	31 (81.6)	0.36	0.06–2.23	0.41	3.83	0.70–21.0	0.18	2.16	0.39–11.8	0.39	0.20	0.02–1.89	0.21	0.41	0.04–3.88	0.65
Group leaders and seniors have helped me to gain a better understanding of migrant workers	36 (94.7)	1.13	0.96–1.33	0.22	0.92	0.83–1.03	1.00	2.60	0.15–45.7	0.50	0.91	0.81–1.04	0.51	0.93	0.84–1.03	1.00
My seniors in CCC have benefited my experience in CCC	35 (94.6)	1.13	0.96–1.325	0.23	0.92	0.82–1.03	1.00	2.50	0.14–44.0	0.51	0.91	0.80–1.04	0.51	0.93	0.83–1.03	1.00
My seniors in CCC are patient and willing to impart their knowledge to me	37 (97.4)	1.06	0.95–1.18	0.47	0.96	0.89–1.04	1.00	0.96	0.89–1.04	1.00	0.96	0.88–1.04	1.00	0.96	0.90–1.04	1.00

### Qualitative interviews

Four themes were identified namely Empathy; Social Awareness & Cultural Competency; CCC Topics and Peer-to-Peer Teaching.

#### Empathy

The topic of empathy was brought up in 2 main ways. Firstly, participants expressed that empathy is contextual. Their interactions with migrant workers provided them the opportunity to appreciate better their lives, especially with respect to the challenges which they experience. This guided the way participants developed their thoughts and the way they eventually interacted with migrant workers.

“*Every time I go down, I’ll be looking at the world from a very different point of views, I’ll try to understand what they are going through. Their lives are totally different from ours. They have to wake up very early in the morning, they have to work for such long hours. Sometimes I try to put myself in their shoes and try to think how I would be like if I were in their position, working for such long hours, not having much freedom. Being away from family and so on.*” – Year 3 Volunteer (Chinese, Female, Completed 2 Cycles)

“*It was only when I went to their dormitory that I realised that their living quarters look very nice on the outside, but is very cramped inside. They are not very well to do. A lot of them really do care about their health. When I see them, I try to empathise. For example, I try to imagine how I would feel like if I am working overseas for my family. Only after that can I imagine that it must be quite hard for them. Also, a lot of the companies hire their own doctors. However, the doctors don't like to give them enough MCs, which means they don't get enough rest.*” – Year 2 Volunteer (Chinese, Female, Completed 1 Cycle)

Secondly, it was brought up that actual interactions developed a different form of empathy as compared to that developed from simulated sessions in a classroom setting. Empathy was even described by a participant as a form of communication in the context of migrant workers, where language served as a barrier at times.

“*It has definitely taught me to be a better listener, not to have expectations before visits and not to have any pre-formed judgments, so that I can take in not just what their medical complaints are but also their stories. If you’re more open and listen more, then you will be able to absorb the migrant mentality better.*” – Year 3 Volunteer (Chinese, Male, Completed 1 Cycle)

“*I was able to demonstrate more empathy. I have more opportunities to demonstrate empathy. It gives me that chance to interact. I think empathy is one of the key aspects that you’ll need in order to communicate with people with such backgrounds because of the language barrier.*” – Year 4 Volunteer (Chinese, Male, Completed 1 Cycle)

#### Social Awareness & Cultural Competency

A number of participants expressed that they had minimal interaction with migrant workers prior to their involvement in CCC. As of consequence, numerous participants expressed that the interactions they had with migrant workers, through CCC, allowed them to correct any potential pre-conceived ideas they may have had with regards to migrant workers, and also understand in greater depth the various issues migrant workers experience from the cultural, social and healthcare perspectives.

Participants also mentioned that through their interactions, there were able to see migrant workers as human beings and not a forgotten group in society. This concept was expressed in various ways, namely the appreciation of stories, challenges, the development of human connections and a special way of communication which transcends language.

“*...it challenged everything that I knew previously, which was nothing much.”* – Year 2 Volunteer (Chinese, Female, Completed 1 Cycle)

“*I got to know the perspectives of these migrant workers. I was able to understand them better, finding out their difficulties and the circumstances they face while working abroad and the challenges that they face. I think that there’s very little opportunity to do so (understand their perspectives) on a daily basis, because we don’t interact with them, this group of people. But, through this programme, I’m able to better understand how they feel about their role here.*” – Year 4 Volunteer (Chinese, Male, Completed 1 Cycle)

“*I guess the first takeaway would be that I’m more exposed to them now because I don’t usually talk to them. The only foreign workers I come into contact with are probably those I meet on the public transport or at the construction sites. I never really get to talk to them. This is a very good chance for me to be exposed to them. I got to know their lives better, what their pay is like, what their working conditions and their living conditions are like.*” – Year 3 Volunteer (Chinese, Female, Completed 2 Cycles)

“*... the whole idea of humanizing a person or a certain stereotype that you have. You can really do that when you actually go and talk to the person and you ask them about their background, about their family, about things that you can identify with in your own life. When you do that, that person actually becomes -- you can see them... as a brother, as a son, as a father. Then that really helps bridge that human connection, which I think is important connection because often…, it’s very easy to lose sight of this.*” – Year 5 Group Leader (Indian, Male, Completed 4 Cycles)

“*Basically, these people... are often forgotten. We don’t really see their day-to-day life. They don’t receive that much media coverage from a certain project but they need help and they’re just right there in the background. You don’t have to go far to look for other underprivileged people, they’re right here.*” – Year 5 Group Leader (Indian, Male, Completed 4 Cycles)

Participants also described that CCC provided them the platform to understand issues that migrant workers may experience as their pass through the healthcare system in Singapore as foreigners. Their interactions influenced their worldview on migrant workers suggesting potentially improved cultural competence when participants eventually become medical doctors and start treating migrant workers independently in various sectors of the Singapore healthcare system.

“*A lot of these migrant workers come to get medical services from community clinics, because they feel that they are unable to get good medical services from their company doctor. This is a very common issue that arises across these migrant workers who attend community clinics. And so in the future, when I treat a migrant worker, when he is very anxious, I won't think that he is trying to get out of work. I know where this anxiousness comes from, why he feels that the company doctor can't relate to him, that his company doctor isn't treating him well.*” – Year 3 Committee Member (Chinese, Male, Completed 2 Cycles)

“*...it has opened my eyes to an area of the medical sector that is lacking. There’s something that we are missing. There’s something that is not being catered to these people in the clinic...*” – Year 3 Volunteer (Indian, Male, Completed 1 Cycle).

CCC Topics: Commitment, Compassion, Care, Communication, Concerns, Continuity.

As a form of evaluation, participants revealed that they did learn more with regards to the core competencies of the CCC programme. They also mentioned that the programme provided them ample opportunity to practice these core competencies in a structured manner.

Apart from that, participants mentioned that CCC allowed them to practice their communication skills with a special group in society which they may not see as commonly in the main clinics and hospitals in Singapore, providing them a unique skillset in overcoming language barriers and growing their confidence in taking a proper clinical and social history despite language barriers.

“*During each session, the handbook states what we are supposed to focus on. For example, the first session was about communication. So before each session, it’s quite useful to think about what I should expect from each session. It also served as a general guide to how the sessions will progress in the future. It made me reflect more about my experiences in CCC because at the end of the day, we were required to consolidate what we learned and to share our experiences with other members or with friends. I thought it was very useful as a means of consolidating my knowledge.*” – Year 3 Group Leader (Chinese, Female, Completed 2 Cycles)

“*Firstly getting over the language barrier, finding ways to explain things in a very simplistic manner, these are things that CCC has taught me. You have to be creative and ask migrant workers questions in a very simple manner...you have to use your hands, and sometimes you have to draw it out on a piece of paper.*” – Year 3 Committee Member (Chinese, Male, Completed 2 Cycles)

“*It's not that because they are different, you cannot communicate with them. (You need to be) more flexible… more fluid in changing your tone and code switching.*” – Year 3 Volunteer (Chinese, Female, Completed 1 Cycle)

“*Being around migrant workers I guess helps you really… you really need to go down to the simplest possible form of communicating. This includes gestures or a smile or breaking down your questions into simple words. You also get to see your fellow juniors and seniors interacting with people, learn from them and teach them as well.*” – Year 3 Volunteer (Indian, Female, Completed 1 Cycle)

“*…CCC has helped build my confidence in trying to build rapport with people whom I meet.*” – Year 4 Volunteer (Chinese, Male, Completed 1 Cycle)

“*I’m more confident in talking to migrant workers now. Before CCC, I never talked to a migrant worker so I don’t really dare to approach them. Now, after speaking to so many of them, they are just like any one of us, so it’s okay to talk to them.*” – Year 3 Volunteer (Chinese, Female, Completed 1 Cycle)

#### Peer-to-peer teaching

Participants talked about the benefits as well as the challenges they experienced with peer-to-peer teaching. In general, medical students viewed CCC as a safe place to ask questions as well as learn new skills and knowledge. CCC also served as a platform for junior medical students to observe how their seniors and peers take a history and communicate with patients, which assisted them as they developed their own styles of history taking and patient communication.

“*They are my seniors, they’ve been through medical school, they have also interacted with people more in a medical setting and most of them have done CCC before so they can draw from their stories, not just medical but also non-medical stories and of course, I gained from that.*” – Year 2 Volunteer (Chinese, Male, Completed 1 Cycle)

“*In CCC, you learn when you see how the seniors interact with the patients, how they go the extra mile.*” – Year 3 Volunteer (Chinese, Female, Completed 2 Cycles)

Senior students also expressed that CCC served as a useful platform to consolidate their knowledge as they taught their juniors. It also provided them the opportunity to practice teaching others. Teaching was also described as a form of learning for senior students.

“*It (CCC) also helps me to consolidate what I learned. It is also a good way to communicate with more juniors as well. As we are all medical students, the teaching is at a level that you’ll be able to understand better. It made me more confident and made me practice more in terms of how to teach my peers and my juniors.*” – Year 3 Volunteer (Chinese, Female, Completed 2 Cycles)

“*I feel that it was very beneficial that the students teach their peers because not only does it help shape your understanding of the community, but of course, it helps to sharpen your own personal medical knowledge, which is ultimately, what we are here to do.*” – Year 3 Volunteer (Chinese, Male, Completed 1 Cycle)

“*The best way to learn is to teach other people. And definitely I can see that when I teach people. I need to know my facts well in order to teach them. Through the act of teaching, I go through content again in my own mind and that helps me to recall information that I would probably have forgotten. It also forces me into a situation where I need to know my content very well. If not, I cannot teach people properly. It has benefited me a lot personally. I can see that it benefited the seniors before me as well.*” Year 3 Volunteer (Chinese, Male, Completed 1.5 Cycles)

“*If you teach, you remember stuff better. I realise that in medical school you learn a lot of content, but you forget a lot. If you teach someone else, you remember it better in your head. Secondly, this mentality of paying it forward is very important. When you go out in society as a doctor and you have more capabilities to do things, you will have to use a similar mindset, beyond the migrant community. So, I guess this really encourages community engagement, in the sense that you pay it forward by using the skills and knowledge that you know to teach other people, and use these skills and knowledge to benefit others.*” – Year 2 Committee Member (Chinese, Female, Completed 2 Cycles)

However, a participant did mention that being requested by a junior to teach did reveal her lack of confidence and knowledge on certain topics. This reveals that the value of peer-to-peer teaching is limited by the extent of knowledge and skills students involved have. The concern on teaching wrong content was revealed as well.

“*When I first started to teach, I was a year 3 medical student, and my own knowledge wasn't the best, so the confidence wasn't there as well. So that is the main challenge because you didn't know whether you were teaching the wrong things.*” – Year 3 Volunteer (Chinese, Female, Completed 2 Cycles)

## Discussion

CCC was initiated with the intention of nurturing empathy and encouraging peer-to-peer teaching amongst medical students. Both the quantitative survey and qualitative interview affirmed improvements in volunteers’ empathy as well as identify components of CCC which contributed to the volunteers’ development.

### Improved empathy and Minimising costs

The benefits from volunteering in CCC described by participants through the FIPSE highlights the extent that CCC succeeds as a service learning initiative. As a student led initiative that leverages and seeks to enhance ongoing community clinics, the reported benefits were achieved at minimal financial (mainly transportation costs borne by participants) and manpower cost. The utilisation of volunteer manpower is consistent with other studies in maintaining low cost while ensuring effectiveness of their programs and benefit for all stakeholders [21, 22]. Similar to findings on medical students hospice volunteers, CCC provides additional benefit to the institution/community clinics as they provide a steady stream of volunteers without the cost of the outreach and community recruitment [23]. Hence, by linking to pre-existing community clinics, CCC is highly replicable in many countries with accessible clinics for both patients and participants. From both the quantitative and qualitative data, there are promising results that support that participation in CCC improved volunteers’ empathy. This is in line with similar studies of structured continuous service learning, which demonstrated that such models can improve empathy (defined by willingness to serve underprivileged communities). For example, in a study by Jones et al., participants’ willingness to serve underprivileged communities increased to from 34 to 70% compared to the batch before them [24]. .We similarly hope that by nurturing empathy, a culture of service can be inculcated and reinforced in our participants.

### Improved FIPSE and communication skills

We compared CCC to other studies- a local service-learning program on health screenings, and another on community medicine in Taiwan [19], in which participants reported marked benefit from the FIPSE Survey instrument ability scale. CCC FIPSE scores are comparable or even higher than another local service learning program that has fared well [15]. This could be due to a CCC’s emphasis on structured reflection, discussion and learning, as other studies conducted reflection on an ad hoc basis.

Participants in CCC also reported increased confidence levels and improved communication skills in talking to strangers and patients. Both this study and the service-learning program in Taiwan attributed the immersive nature of exposure, interaction with their communities and structured tasks as key reasons for improved communication skills. Communication, essential in the therapeutic relationship, increases patient satisfaction [25]. However, populations with limited English-speaking abilities, can receive compromised care because of English-predominant conversation, highlighting healthcare providers’ decreased awareness of the language barrier [26]. CCC was able to overcome such barriers as volunteers tailored verbal and non-verbal communications toward the migrant population.

### Improved cultural competency

Cultural competence has been defined as “the ability of individuals to establish effective interpersonal and working relationships that supersede cultural differences”, and a “culturally competent healthcare system” is one that takes into account the importance of culture, health-seeking attitudes and behaviours, to tailor healthcare services to serve the unique needs of the population. [31] In this study, CCC provided opportunities to develop an in-depth understanding of the unique challenges migrant workers perceive they face simply because of their identity. For example our study identified that some migrant workers feel that the medical care received from their company doctors tend to be of a lower standard, particularly as they perceive that these healthcare workers tend to have a view that they are merely seeking medical services to get time off work. Our volunteers also learnt that company doctors are also more hesitant to provide medical certificates to these migrant workers. It also sheds light into the particular problems migrant workers face by virtue of their socio-economic status and limited access to alternative avenues of healthcare. Through these insights, our volunteers are then able to tailor healthcare to these community of migrant workers in future by being aware of their own pre-conceived notions about migrant workers’ health-seeking behavior, and learn not to discount the healthcare complaints migrant workers bring to a consultation room.

### Improved empathy

Participants also feel that CCC allowed them to practice empathy realistically. In many medical schools, students practice empathy with Simulated Patients (SP). By allowing students to interact with migrant workers, it bridges the gap between theory and practice. Empathy is not only positively associated with clinical competence [27], it also improves patient outcomes. Participants in CCC felt that from this exposure to an underprivileged population, they were more accepting and understanding of the migrant workers, and more aligned with the needs of the community. By forming bonds on the ground and emphasizing the similarities rather than differences between the volunteers and the migrant workers, this could de-emphasize a sense of self-importance and build connection and understanding, which would greatly aid in developing empathy. Additionally, many participants were inspired by the seniors’ teaching and sharing of their own experiences. This demonstrates that peer-to-peer teaching and learning can possibly also play a role in boosting empathy in medical students.

### Improved social awareness

Participants in CCC have also developed a greater awareness of how social situations affect health. This was probably achieved through 2 mechanisms: a focus on social history taking in CCC, and an immersive environment of seeing first-hand the living conditions of these migrant workers and its impact on their health. Many participants have reflected that the migrant workers require a more personalised process of medical treatment, tailored to their social background and limitations on their lifestyles. A study in Germany by Keifenheim of 42 medical students in a peer-assisted history-taking course showed that interaction with a real patient, and history taking resulted in marked improvement in identifying and dealing with their emotional and social issues [28]. In our study, participants reflected for the need to have a deeper understanding and empathy for migrant workers, especially in the standard medical care system (eg. in hospitals and polyclinics), given their unique socio-economic circumstance. Both Keifenheim‘s paper and CCC has shown that the two factors of having an immersive experience with a real patient, and a focus on social history taking has led to a greater awareness of social issues in relation to health.

Additionally, CCC has also shown to develop greater awareness of other underprivileged communities and deepen the participants’ understanding of society and varying worldviews. This was mainly achieved through allowing the participants to interact with the migrant community in a safe and natural environment, where both the participant and the migrant worker are both very willing to share their experiences with each other. The reflection participants conducted at the end of the session and in their own personal time also contributed to the development of a greater awareness of the community and helped them translate it into greater awareness of other communities. Similarly, in a Pittsburgh study of occupational therapy students participating in a service learning project with marginalised communities, students who were given the opportunity to interact with and learn from an underprivileged community in a natural context were able to gain a deeper understanding of the community they served and practice it within and beyond the community [29]. Although this project had a longer time frame, and more sessions with the community they served, the factors identified contributing to the development of a larger worldview was that of the sharing of experiences by the community and the natural context the project was set in. This is similar to the experience the participants had at CCC. This will serve the participants well when they encounter other underprivileged communities in the future, as students or as medical professionals. Patients will also benefit from dealing with healthcare professionals that have a deeper awareness of their situation, and hence be rendered more personalised and effective care.

### Peer-to-peer teaching

Participants felt positively about peer-to-peer teaching and many felt this was a safe environment to do so. Seniors benefited by being able to consolidate their knowledge, add meaning to their experiences and clarify their own understanding. Juniors enhanced their knowledge and understanding though thematic and opportunistic discussions. Peer-to-peer teaching not only enhanced senior-junior interaction, but the learning of both parties involved. In a systematic review paper of peer-to-peer teaching in clinical education [29], it was found to be an effective educational intervention that aids learning. In our study, some seniors reported a sense of responsibility towards teaching their juniors when opportunities were presented to them, and juniors felt more motivated to teach their peers in the future. This creates a culture of collaboration and sharing of knowledge, enhancing each student’s learning experience.

### Limitations of study

There were certain limitations to this study. The sample size of our study population was small for the quantitative aspects of our study. This was largely a result of both the number of students who participated in CCC programme and the attendance at the last session when we carried out the survey. While efforts are taken to avoid planning sessions during examination periods, the last sessions of the CCC programme unfortunately tends to coincide with the period of time leading to the medical students examinations where students may focus more on studies than extra-curricular activities.

Unfortunately, we are unable to assess the views of participants that missed their last session of CCC. It may be possible that such students who prioritised exam preparation over their last session may not have felt they have benefited as much. Anecdotally, however, many participants who completed one cycle of CCC, both those who attended and did not attend their last cycle, do continue to volunteer with CCC adhoc or in the next cycle.

Sustainability is another vital aspect for future research, aptly raised by a commentary on a student-led initiative in the University of California, San Francisco [18]. If CCC is to be sustainable, it must endure generations of handing-over and leadership transitions and continue to recruit volunteers of equal passion. Along with this test of time, it may be useful to study if volunteers continue to serve migrant or other underserved communities as they progress in their medical career.

## Conclusion

In conclusion, majority of participants in CCC programme believed that peer-to-peer teaching enhanced learning for both seniors and juniors. Given that CCC focused primarily on serving the foreign worker population in Singapore, we also found that the majority of participants reported an improvement in soft skills such as empathy and communication skills, as the experience allowed for a deeper understanding and wider exposure towards underprivileged populations. Thus, we highly encourage further studies to be conducted to explore the efficacy of such an educational model applied across different target service populations, and hope that this model can then be replicated in other educational institutions to enhance and boost learning beyond the classroom.

## Additional files

Additional file 1: CCC Quantitative survey. (DOCX 19 kb)

Additional file 2: Interview Guide for Constructing Care Collaboration Volunteer Interview. (DOCX 16 kb)

## Data Availability

The datasets used and analysed during the current study are available from the corresponding author on reasonable request.

## Abbreviations

CCC: Constructing Care Collaboration
FIPSE: Fund for the Improvement of Postsecondary Education
NGOs: Non-Governmental Organizations
NUS Medicine: Yong Loo Lin School of Medicine, National University of Singapore
SP: Simulated Patients
