# Supplementary material for: Evaluation of Constructing Care Collaboration - nurturing empathy and peer-to-peer learning in medical students who participate in voluntary structured service learning programmes for migrant workers
Source: BMC Med Educ. 2019 Aug 8;19:304. doi: 10.1186/s12909-019-1740-6 (PMC6686532; doi:10.1186/s12909-019-1740-6)
Supplement: Supplementary file 1 — CCC Quantitative survey. (DOCX 19 kb) [file 12909_2019_1740_MOESM1_ESM.docx]

## Additional file 1: CCC Quantitative survey

**Constructing Care Collaboration Survey**

(i) Gender:   M  / F

(ii) Medical Year:     1  / 2  / 3 / 4 / 5

(iii) Age: _________

(iv) Ethnicity: Chinese/ Malay/ Indian/ Others:_________ (Please specify)

(v) Number of cycles attended so far: 1  / 2 /  3 / 4 /  5 / 6

(vi) Number of CCC Sessions attended (each cycle has 6 sessions):  ________ (Please specify Number)

(vii) Current clinic attended: Penjuru Clinic / Caring Community Clinic / Both

(viii) My roles in CCC include:

A. Participant: ______ (Specify year)

B. Group Leader: ______ (Specify year)

C. Committee Member: ______ (Specify year)

(ix) Please read the statements below and grade as:

1: Agree

2: Unsure but tend to agree

3: Unsure but tend to disagree

4: Disagree

5: Do not understand the statement

Volunteering in CCC has helped me to:

| (1) Feel more responsible for others in the Community | 1 | 2 | 3 | 4 | 5 |
| --- | --- | --- | --- | --- | --- |
| (2) Think more of others | 1 | 2 | 3 | 4 | 5 |
| (3) Think more about the future | 1 | 2 | 3 | 4 | 5 |
| (4) Improve my leadership skills | 1 | 2 | 3 | 4 | 5 |
| (5) Participate in community affairs | 1 | 2 | 3 | 4 | 5 |
| (6) Take more action | 1 | 2 | 3 | 4 | 5 |
| (7) Appreciate teamwork and co-operation among peers | 1 | 2 | 3 | 4 | 5 |
| (8) Further develop communication, listening and negotiation skills | 1 | 2 | 3 | 4 | 5 |
| (9) Respect different opinions | 1 | 2 | 3 | 4 | 5 |
| (10) Feel more responsible for others in the Community | 1 | 2 | 3 | 4 | 5 |
| (11) Be more tolerant of different people | 1 | 2 | 3 | 4 | 5 |
| (12) Compromise | 1 | 2 | 3 | 4 | 5 |
| (13) Build confidence & take on new responsibilities | 1 | 2 | 3 | 4 | 5 |
| (14) Think more critically | 1 | 2 | 3 | 4 | 5 |
| (15) Apply what I learnt in medical school | 1 | 2 | 3 | 4 | 5 |
| (16) Improve my medical knowledge | 1 | 2 | 3 | 4 | 5 |
| (17) Improve my clinical diagnostic skills | 1 | 2 | 3 | 4 | 5 |
| (18) Better Comprehend the moral and ethical issues in health care | 1 | 2 | 3 | 4 | 5 |
| (19) Better Identify social issues and concerns | 1 | 2 | 3 | 4 | 5 |
| (20) Enhance my understanding of the use of public health measures in resource-poor settings | 1 | 2 | 3 | 4 | 5 |
| (21) Appreciate and identify better gaps or deficiencies in the healthcare system | 1 | 2 | 3 | 4 | 5 |
| (22) Better Appreciate my own health, living condition | 1 | 2 | 3 | 4 | 5 |
| (23) Increase my confidence in approaching migrant workers | 1 | 2 | 3 | 4 | 5 |
| (24) Increase my confidence in talking to people from different social backgrounds. | 1 | 2 | 3 | 4 | 5 |
| (25) Increase my confidence in teaching my peers | 1 | 2 | 3 | 4 | 5 |
| (26) Increase my knowledge from what was taught by my peers during CCC | 1 | 2 | 3 | 4 | 5 |
| (27) I feel my experience in peer to peer teaching at CCC has enabled me to learn more from similar modes of peer-to-peer teaching (outside of CCC) | 1 | 2 | 3 | 4 | 5 |
| (28) I feel that the Group leaders and seniors have helped me to gain a better understanding of Migrant workers | 1 | 2 | 3 | 4 | 5 |
| (29) I feel that my seniors in CCC have benefited my experience in CCC | 1 | 2 | 3 | 4 | 5 |
| (30) I feel that my seniors in CCC are patient and willing to impart their knowledge to me | 1 | 2 | 3 | 4 | 5 |

(vi) Other Comments (Please cite specific examples):

___________________________________________________________________

___________________________________________________________________

(vii) Are you involved in other ad hoc/long term volunteering activities (Please list):
