# Supplementary material for: Evaluation of Constructing Care Collaboration - nurturing empathy and peer-to-peer learning in medical students who participate in voluntary structured service learning programmes for migrant workers
Source: BMC Med Educ. 2019 Aug 8;19:304. doi: 10.1186/s12909-019-1740-6 (PMC6686532; doi:10.1186/s12909-019-1740-6)
Supplement: Supplementary file 2 — Interview Guide for Constructing Care Collaboration Volunteer Interview. (DOCX 16 kb) [file 12909_2019_1740_MOESM2_ESM.docx]

## Additional file 2: Interview Guide for Constructing Care Collaboration Volunteer Interview

Before beginning the interview, please explain the information in the *participant information sheet* to the interviewees, and obtain written consent (via the *consent form*).

Additional Points of Clarification:

●      The purpose of the study: The purpose of this research is to evaluate the Constructing Care Collaboration as an avenue for nurturing empathy and in encouraging peer-to-peer teaching in Medical Students.

●      Why they are eligible to be interviewed: Interviewees are Medical Students and have experienced at least one cycle of CCC

●      How the interview will be conducted: They will be asked a series of questions. All interviews will be digitally audio-recorded for the purpose of ad-verbatim transcription. Where volunteers do not allow audio recording, interviewers will take down field notes instead.

●      How their privacy will be protected: No identifiers will be recorded. Digital data will be stored on password-protected computers.

●      At any time if the volunteer wishes to not participate, they may do so without any consequence and any data already taken will be discarded.

●      If the participant is unwilling to be audio-recorded, investigators will proceed on and take field notes instead.

Order of interview questions

General Questions:

1. How many cycles have you been involved in CCC for?

2. Where were you posted to for your CCC cycles?

3. What is your current role in CCC? (Volunteer/ Group Leader/ Committee Member)

4. How active have you been involved in CCC? (to estimate percentage attendance for clinic sessions)

5. Why did you join CCC?

6. How did you find your experience at CCC? Could you tell me what a typical CCC Session is like? (deleted previous question 8, modified question 7)

7. What are your 3 greatest takeaways from CCC?

8. What have you learnt from these takeaways?

9. Would you recommend this program (CCC) to your fellow medical students? Why or Why not?

10.  Would you be interested in attending another cycle of CCC? Why/ Why not?

a.    If you were interested to attend another cycle of CCC, would you want to attend it in the same capacity (Group Leader/ Volunteer/ Committee Member etc)

Empathy:

11.  What do you understand by the term empathy?

12.  Did this understanding differ from before you started CCC?

a.    If yes, in what way did your understanding of empathy differ from before you started CCC?

i.  Do you attribute this change to CCC or any other event?

b.    If no, why not?

13.  Could you describe a situation in which you displayed empathy (within CCC)?

Social Awareness/ Cultural Competency:

14.   What was your understanding of the situation of migrant workers before CCC?

15.   How has that understanding changed since your participation in CCC?

16.  Do you feel CCC has changed your preparedness/confidence in talking to/relating to migrant workers?

17.  If yes, how has CCC changed your preparedness/confidence in talking to/relating to migrant workers?

a.    If no, why not?

18.  Do you feel CCC has assisted you in understanding not just the migrant community but people who are different from you? Explain.

19.  Do you feel CCC has assisted you in understanding not just the migrant community but individuals from other needy and underprivileged communities? Explain.

CCC Topics: Commitment, Compassion, Care, Communication, Concerns, Continuity:

20.  Do you find journaling within the CCC Handbook useful? Explain.

21.  Do you find the discussions about the topics (as stated in the CCC handbook) useful? Explain.

22.  How confident were you to discuss medical issues with patients before joining CCC?

a.    In what ways has joining CCC changed your confidence level?

23.  How confident were you to discuss non-medical issues with patients before joining CCC?

a.    In what ways has joining CCC changed your confidence level?

24.  Did you encounter any challenges in keeping up with the weekly teachings? Explain.

25.  Do you feel that your level of communication skills has changed after CCC? If so, how has it changed?

Encouraging/ Confidence in/ Positive gains from Peer-to-peer teaching:

26.  Have you had the opportunity to teach your fellow peers during CCC?

27.  Have you been taught by your fellow peers during CCC?

28.  Do you think it is beneficial for medical students to teach their fellow peers? Explain.

29.  Do you think it is beneficial for medical students to be taught by their fellow peers? Explain.

30.  How well prepared were you to teach your peers before CCC?

31.  Has CCC improved your confidence in teaching your fellow peers?

32.  Do you feel that CCC has influenced your desire to teach your fellow peers?

33.  Did you encounter any challenges while you were teaching your peers? Please elaborate.

34.  Did you encounter any challenges when being taught by your peers? Please elaborate.

35.  Are there any ways to improve the peer-to-peer teaching process in CCC?

36.  In what ways has CCC benefitted your academics within medical school?

37.  In what ways has CCC benefitted your clinical skills within medical school?

38.  In what ways has CCC benefitted your communication with patients in medical school, if relevant?

Closing Remarks

39.  What are some ways in which we can improve CCC?

40.  If you were provided the opportunity to initiate a project for migrant workers, what would it be?

41.  Do you have anything else to add?
